# De novo mast cell leukemia without CD25 expression and KIT mutations: a rare case report in a 13-year-old child

**DOI:** 10.1186/s13000-018-0691-2

**Published:** 2018-02-20

**Authors:** Yalin Zheng, Lin Nong, Li Liang, Wei Wang, Ting Li

**Affiliations:** Department of Pathology, Peking University First Hospital, 8 Xishiku Street, Xicheng District, Beijing, 100034 China

**Keywords:** Systemic mastocytosis, Mast cell leukemia, Tryptase, CD25, KIT

## Abstract

**Background:**

Mast cell leukemia (MCL) is a very rare form of systemic mastocytosis (SM) and accounts for less than 0.5% of all mastocytosis. The diagnosis of MCL requires the presence of SM criteria, accompanied by leukemic infiltrating of atypical mast cells (MCs) in bone marrow (BM), peripheral blood as well as extracutaneous organs. MCL is a fatal disease that almost always behaves aggressively, and the median survival time is only about six months. Herein, we present a rare case of de novo MCL without CD25 expression and KIT mutations.

**Case presentation:**

A previously healthy 13-year-old boy was referred to our hospital due to incidental discovery of an enlarged right tonsil. Diffuse infiltration of medium-sized hematopoietic blasts was found in his right tonsil, BM and multiple lymph nodes. The neoplastic cell population was subsequently revealed to exhibit differentiation towards the mast cell lineage by expressing CD117 and tryptase, but the cell population lacked expression of CD25/CD2 and the activating mutation of the KIT gene. An abnormal karyotype was identified, but no leukemia-associated fusion genes were found. Involvement of peripheral blood, bone and lung was subsequently demonstrated. The most important differential diagnosis included tryptase-positive (T+) acute myeloid leukemia, myelomastocytic leukemia and basophilic leukemia. The morphological characteristics and infiltrating patterns of the abnormal MCs supported the final diagnosis of MCL. Although intensive chemotherapy and allogeneic stem cell transplants were performed on the patient, he died 18 months after initial presentation.

**Conclusion:**

Due to its rarity, the diagnosis of MCL without typical immunophenotype and genetic aberrations is particularly challenging. Comprehensive investigation of clinical and pathological features to exclude other T+ myeloid neoplasms is necessary.

**Electronic supplementary material:**

The online version of this article (10.1186/s13000-018-0691-2) contains supplementary material, which is available to authorized users.

## Background

Mast cells (MCs) are defined as a type of leukocytes derived from hematopoietic stem cells, which contain abundant cytoplasmic basophilic granules rich in various mediators, such as serine proteases, and play a key role in innate and adaptive immunity [1]. Mastocytosis, the most well-known disease derived from MCs, presents as a heterogeneous group of diseases characterized by clonal proliferation of MCs in various organ systems. In the revised 2016 WHO classification, mastocytosis was defined as a separate disease category and is no longer listed under the heading of myeloid proliferative neoplasms. According to its clinical and pathologic features, mastocytosis is classified into cutaneous mastocytosis, systemic mastocytosis (SM) and mast cell sarcoma [2–6].

Mast cell leukemia (MCL), one of the rarest types of leukemia, is the leukemic form of SM that accounts for less than 0.5% of all mastocytosis. There are only approximately 70 well-documented cases reported to date [5]. The diagnostic criteria of MCL has been clearly defined in the 2008 WHO and the most recent revised 2016 WHO classification [2–4]. Morphologically, atypical MCs with a dense and diffuse infiltration pattern are shown in the bone marrow (BM) and one or more extracutaneous organs. The neoplastic MCs usually express tryptase, CD117 (KIT) and CD25, with co-expression of CD2 in a proportion of cases. Mutations of the KIT proto-oncogene have been widely reported in human mastocytosis. In MCL cases, mutations in exon 8, 9, 10, 11, 13 and 17 have been previously described [6]. MCL may occur de novo or secondary to SM. The ratio of de novo cases to secondary cases reported was approximately 3:1 [6]. MCL usually behaves aggressively with poor responses to current treatments. We report an unusual case of de novo leukemic MCL without expression of CD25 and KIT mutations, which initially raised the suspicion of tryptase-positive (T+) acute myeloid leukemia (T+ AML) or myelomastocytic leukemia (MML).

## Case presentation

**Fig. 1:**
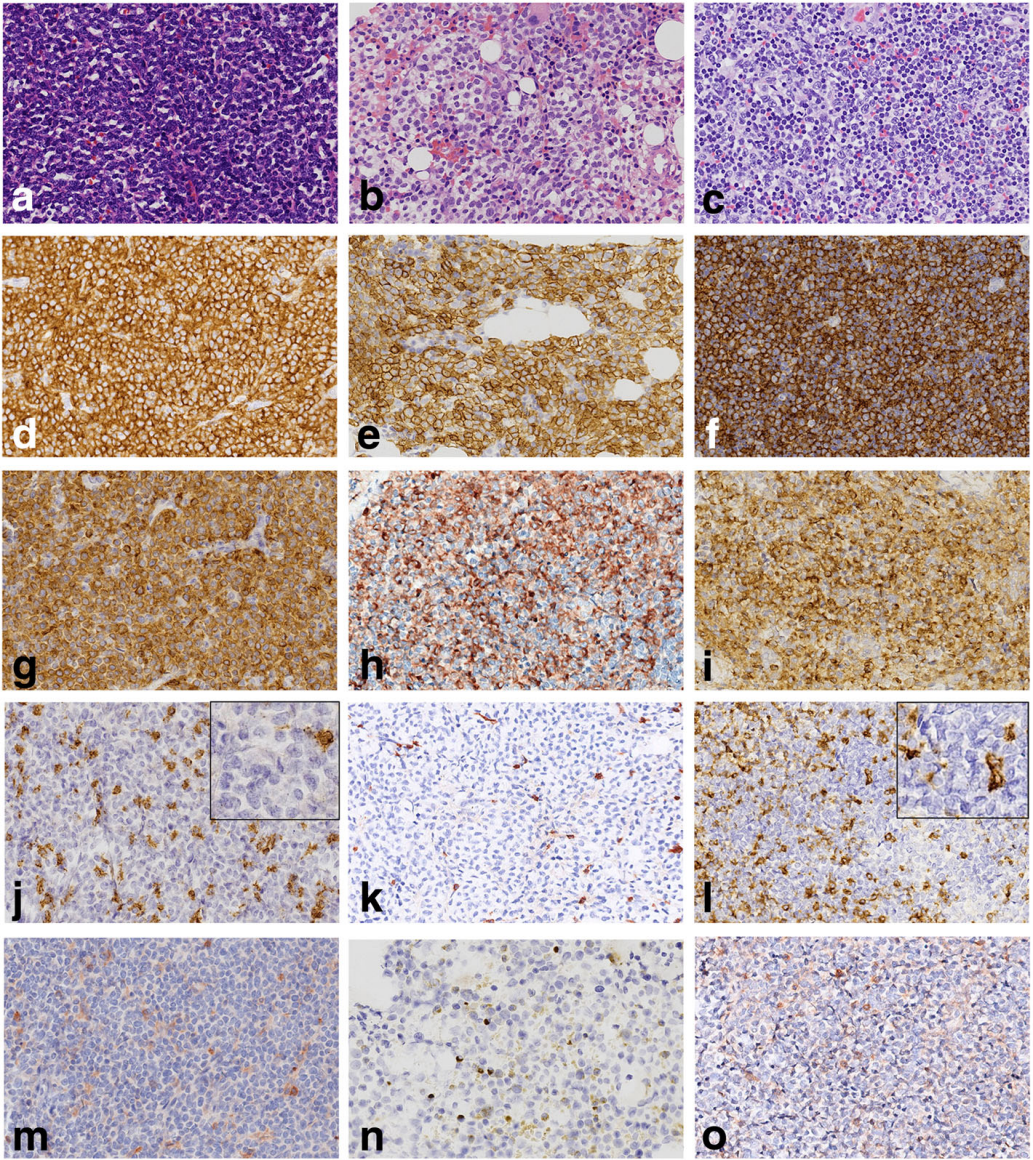
Morphologic and immunohistochemical features of the case. **a** Tonsil biopsy showed the architecture was diffusely effaced by proliferation of medium-sized hematopoietic blasts. Immunohistochemically, the neoplastic cells were positive for (**d**) CD117 and (**g**) tryptase but almost negative for (**j**) CD2 and (**m**) CD25. The (**b**) BM trephine biopsy and (**c**) cervical lymph node biopsy shared similar morphological and immunohistochemical features as the neoplastic cells in the tonsil, but the atypical cells in the BM biopsy showed differentiating features similar to immature MCs (**b**). **d**-**f** CD117 staining in the tonsil, BM and lymph node, respectively. **g**-**i** Tryptase staining in the tonsil, BM and lymph node, respectively. **j-l** CD2 staining in the tonsil, BM and lymph node, respectively. **m**-**o** CD25 staining in the tonsil, BM and lymph node, respectively. **a**-**c** Hematoxylin and eosin, 400×. **d**-**o** Immunohistochemistry, 400×

**Fig. 2:**
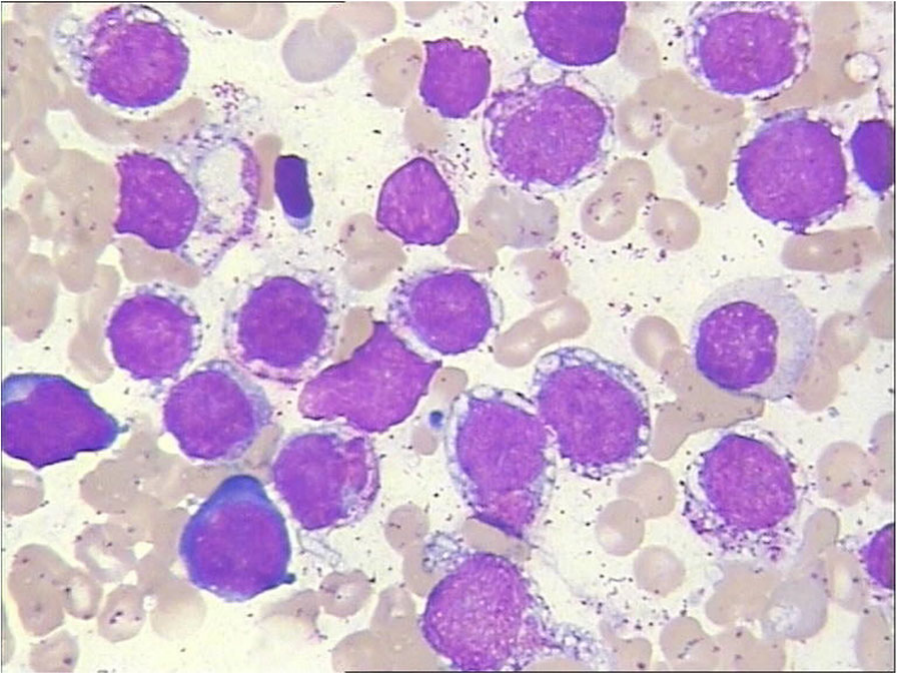
Wright-Giemsa-stained bone marrow smear showed numerous metachromatic blast cells with vacuolated cytoplasm containing varying numbers of metachromatic granules (1000×)

A 13-year-old boy with no symptoms was incidently  found to have an enlarged of right tonsil during an orthodontic procedure. The patient was previously healthy, except for a one-year history of psoriasis. Physical examination showed the right tonsil was obviously enlarged with a multinodular appearance and purulent exudate. Mild splenomegaly was found by palpation and computed tomography (CT). The superficial lymph nodes were not palpable. Laboratory examination was unremarkable. Then, a tonsillectomy was performed. Histological examination revealed a diffuse proliferation of monomorphic, medium-sized cells that partially effaced the architecture. The cells resembled hematopoietic blasts, with finely dispersed chromatin, moderate vacuolated cytoplasm, medium-large sized round nuclei and indistinct nucleoli. Mitotic figures were easily found. Neither angiodestruction nor necrosis was observed. Immunohistochemical studies showed positive immunoreactivity of the cells for CD45, CD43, tryptase, CD117, CD56 and negative immunoreactivity for CD34, TdT, CD10, CD20, CD3, PAX5, CD68(KP1), MPO, CD30, CD2, CD7, CD25, TIA1, GranzymeB, CD123, CD235 and CD61 (Fig. 1, Table 1). The Ki67 labelling index of the cells was greater than 60%. In situ hybridization for Epstein-Barr virus (EBV) encoding RNA (EBER) was negative. BM trephine biopsy and aspirate smears were obtained immediately after the abnormality was found. The BM biopsy showed the normal architecture was almost completely replaced by atypical cells that shared similar immunohistochemical and molecular characteristics with the blasts in tonsils but shared differentiating features with immature MCs, such as medium-sized oval or spindle shaped nuclei. The BM aspirate smears were also found to consist of an excess of atypical blasts with basophilic cytoplasm containing varying numbers of metachromatic granules, accounting for 69.5% of all nucleated BM cells (Fig. 2). Auer rods were absent. These atypical cells were positive for HLA-DR, CD56, CD33, CD117, BDCA-1, CD9, CD69, CD11c, and CD11b but were negative for CD34, CD123, CD25, CD2 and MPO by flow cytometry. Chromosome analysis of BM showed an abnormal karyotype of 47, XY, + 5, t (1; 9) with an equivocal translocation involving chromosomes 1 and 9. None of the 41 leukemia-related fusion genes we detected by real-time PCR were positive. The peripheral blood smear showed up to 16% abnormal cells, which were confirmed by flow cytometry to be of similar immunophenotype as those in BM. Involvement of multiple lymph nodes, the right scapula and lung was also detected by PET-CT, and the involvement of lymph nodes was confirmed by biopsy. Unfortunately, the serum tryptase level was unavailable due to technical limitations. The whole picture was in line with a myeloid neoplasm with mast cell differentiation. Sequencing of exon 8 (p. 412–448), exon 9 (p. 449–513), exon 11 (p. 549–591), exon 13 (p. 628–663), and exon 17 (p. 788–828) of the KIT gene revealed no detectable mutation, including the most frequently detected KIT D816V mutation. However, the diagnosis of leukemic variant of MCL was made, based on the typical histopathological characters and specific immunostaining of the abnormal MCs. Materials and methods are shown in more details in an additional file [see Additional file 1: Materials and Methods].

**Table 1:** Comparison of key markers between our case and related diseases

Markers	Our case				
	Tonsil/BM/LN	MCL/ASM	MML	AML	BAL
CD117	+	+	+	+	–
Tryptase	+	+	+	−/+	−/+
CD2	–	+	–	–	–
CD25	**–**	+	–	–	+/−
CD34	–	–	–	+	+
CD68(KP1)	–	–	−/+	+	+
MPO	–	–	−/+	+	+
CD56	+	–	−/+	+	–
TdT	–	–	−/+	−/+	–
c-kit D816V	–	+	–	–	–

*Abbreviation*: *BM* bone marrow, *LN* lymph node, *MCL* mast cell leukemia, *ASM* aggressive systemic mastocytosis, *MML* myelomastocytic leukemia, *AML* acute myeloid leukemia, *BAL* basophilic leukemia

The patient underwent 3 cycles of combination chemotherapy, including fludarabine, cytosine arabinoside, dasatinib and daunorubicin. Then, an allogeneic bone marrow transplant was performed. Complete remission was achieved; however, the patient relapsed 7 months later. Rapidly, he suffered from tumor dissemination, and finally died from cerebral hemorrhage almost 18 months after initial presentation.

## Discussion

MCL is one of the rarest types of leukemia, first described by Joachim in 1906 [7]. According to the 2008 WHO and the most recent revised 2016 WHO classification, MCL is diagnosed when criteria for SM are fulfilled and when BM biopsy shows leukemic infiltration by atypical/immature MCs, with increasing atypical MCs in the BM smears (> 20% of all nucleated cells) and/or in the peripheral blood (> 10% of all leukocytes in typical cases) [2–4]. An “aleukemic” MCL is defined if the circulating MCs are less than 10%. The diagnosis of SM can be made when the major criterion and one minor criterion are present or when at least three minor criteria are present. Major criteria include the following: multifocal dense infiltrates of MCs (> 15 MCs in aggregate) in BM sections and/or other extracutaneous organ(s). Minor criteria include the following: (1) > 25% MCs in BM infiltrates, infiltrates in another extracutaneous organ or in a BM smear showing spindle-shaped or atypical morphology; (2) KIT mutation at codon 816 in extracutaneous organs; (3) BM MCs express CD2 and/or CD25 by flow or IHC; (4) serum total tryptase > 20 ng/mL [2–4].

The case we report herein is characterized by diffuse infiltration of atypical cells in BM, tonsil and lymph nodes. The morphology of metachromatic blasts in BM smears and positivity for CD117 and tryptase revealed the origin of the mast cell lineage. The diagnosis of MCL was made as the case had fulfilled the major criterion (dense infiltrates of aggregated MCs in more than one extracutaneous organ systems) and one minor criterion (> 25% of atypical MCs in BM biopsy and/or smears) for systemic mastocytosis, with up to 16% atypical MCs in peripheral blood smears. The case did not meet other minor criteria including expression of CD25 and/or CD2, an activating point mutation at codon 816 of KIT, and the serum tryptase level could not be assessed due to technical limitations. However, MCs in MCL may lack expression of CD25 and CD2 in a proportion of MCLs, and they have been reported to be double negative for CD2/CD25 in 38% of MCL cases [6, 8]. Although previous studies have shown that nearly 50% of de novo MCLs had the KIT D816V mutation and have reported other non-D816V mutations at exon 8, 9, 10, 11, 13, and 17 of KIT, there were no KIT mutations found in a subset of MCLs [6, 9]. Sequencing of exon 8, 9, 11, 13 and 17 of the KIT gene in our patient revealed no detectable mutation. Although almost all mutations ever reported in mastocytosis have been covered, it is difficult to exclude other possible mutations in the KIT gene unless the entire KIT gene is sequenced.

Based on the unusual manifestations of our case, the most important differential diagnosis is T+ AML. The compact round blast-like immature cells with expression of CD43, CD33, CD117 and CD56, absence of CD25 and CD2 expression, as well as lack of a KIT mutation in this case, would be consistent with an early stage of myeloid lineage differentiation. Although the expression of tryptase is thought to be specific for mast cells, the existence of T+ AML is reported to be not uncommon according to the published studies [10, 11]. T+ AML represents a rare variant of AML that coexpressed tryptase (but not CD117) in CD34+ myeloblasts, usually with FAB classification M0 or M1. Sperr et al. hypothesized that T+ AMLs arise from a leukemic progenitor that exhibits a limited potential to differentiate into mast cells and/or basophils [10]. However, the immunophenotype of CD34-/CD117+ in our case revealed the blasts belong to the MC lineage, and metachromatic blasts in BM smears were rarely seen in T+ AML [12].

MML is another important differential diagnosis of SM, especially when systemic mastocytosis with an associated hematological neoplasm or MCL with a lack of KIT codon 816 mutations is suspected. MML is an extremely rare and underrecognized myeloid neoplasm belonging to the group of T+ myeloid neoplasms and shares overlapping features with MCL, T+ AML and basophilic leukemia (BL) [12]. Although MML has not been included in either the WHO or the FAB classification systems of myeloid and lymphoid neoplasms so far, consensus has been reached on the diagnostic criteria of MML [9]. It is characterized by prominent atypical MCs or metachromatic blasts exhibiting differentiation into the MC lineage in an advanced myeloid neoplasm, but it does not fulfil the criteria for SM. Positivity for mast cell-related antigens, such as tryptase and CD117(KIT), reveals the nature of the neoplastic cells as mast cell precursors, while expression of CD25 and CD2 was absent. None of the specific (recurrent) molecular or cytogenetic aberrations, especially the specific point mutations at codon 816 of KIT in SM could be detected in MML [9, 12, 13]. In addition, MML always shows diffuse interstitial infiltration of mast cells in tissues but never meets the major diagnostic criterion of SM (≥15 MCs aggregates) or exhibits diffuse-compact infiltration, as in our present case. The absence of evidence of simultaneous, advanced, myeloid neoplasms would also exclude the diagnosis of MML. It is reported that the leukemic cells in a group of patients with AML (approximately 40%) produce significant amounts of tryptase with subsequent elevated serum tryptase levels, indicating the involvement of a mast cell lineage [10, 11].

Another disease that should be ruled out is BL. Mast cells share a number of similar antigens and functional properties with basophils. Basophil granules containing histamine and heparin can be seen in either basophils or MCs. Both cells can release histamine upon binding to immunoglobulin E. However, combined CD117 and tryptase staining is a key feature of MCs that distinguishes them from basophils, as well as other mast cell-associated disorders [10]. Tryptases are lineage-associated serine proteases primarily expressed in MCs [10] and have a significantly lower amount in blood basophils, while all other leukocytes are tryptase-negative under physiologic condition.

MCLs usually show an aggressive clinical course with a median survival time of no more than 6 months, and there is no approved standard therapy, to date. The patient died 18 months after initial diagnosis, even with aggressive chemotherapy and bone marrow transplant. Due to the rarity of MCL, few options are available for treatment, a situation requiring more clinical trials to explore novel regimens.

## Conclusion

In summary, herein, we present an unusual case of de novo MCL without CD25 expression and KIT mutations, with an aggressive clinical course and poor prognosis. The diagnosis of MCL is challenging when there are a lack of typical immunophenotypes and genetic aberrations, and the serum tryptase level is unavailable due to technical limitations. Under the circumstances, the characteristic morphology of MCL is the most important evidence that led our diagnosis in the right direction. New potential molecular aberrations in such cases need to be further investigated.

## Additional file

Additional file 1: Materials and Methods. (DOCX 22 kb)

## Abbreviations

AML: Acute myeloid leukemia
BL: Basophilic leukemia
BM: Bone marrow
CT: Computed tomography
EBV: Epstein-Barr virus (EBV)
MCL: Mast cell leukemia
MCs: Mast cells
MML: Myelomastocytic leukemia
SM: Systemic mastocytosis
T +: Tryptase-positive
