# Supplementary material for: De novo mast cell leukemia without CD25 expression and KIT mutations: a rare case report in a 13-year-old child
Source: Diagn Pathol. 2018 Feb 20;13:14. doi: 10.1186/s13000-018-0691-2 (PMC5819157; doi:10.1186/s13000-018-0691-2)
Supplement: Supplementary file 1 — Materials and Methods. (DOCX 22 kb) [file 13000_2018_691_MOESM1_ESM.docx]

**Additional file**

**Materials and Methods**

1. **Histopathology and Immunohistochemistry**

Tissue specimens were fixed in 10% formalin or neutral-buffered formalin (bone marrow biopsy was decalcified by 5% nitric acid for 10 minutes), routinely processed, and embedded in paraffin. Sections of 4-μm thickness were stained with H&E. Immunohistochemical staining was performed using a DAKO EnVison ^TM^ detection kit (DAKO, Denmark). The tissue sections underwent heat-induced antigen retrieval in citrate acid buffer (pH 6.0) or EDTA-Tris (pH 9.0). Antibodies were used against each specimen, including CD45 (DAKO, clones 2B11+PD7/26), CD43 (Novocastra, clone MT1), tryptase (Santa Cruz, sc-33676), CD117(DAKO, polyclonal KIT), CD56 (Zymed, clone 123C3), CD34 (Immunotech, clone QBEnd/10), TdT (Zymed, clone SEN28), CD10 (Novocastra, clone56C6), CD20(DAKO, clone L26), CD3 (DAKO, clone Ps1), PAX5 (Zhongshan, clone EP156), CD68(Abcam, clone KP1), MPO (DAKO, human anti-MPO), CD30(DAKO, clone BER-H2), CD2 (Abcam, ab206089), CD7 (Novocastra, CD7-272), CD25 (Vector, clone 4C9), TIA1((Novocastra, clone TIA1), GranzymeB (Novocastra, clone 11F1), CD123 (BD Biosciences, clone 7G3), CD235 (DAKO, clone JC159)and CD61 (Roche, clone 2f2). Antibodies were considered positive when more than 50% of the tumor cells showed immunopositivity.

1. **In Situ Hybridization (ISH) Test for Epstein-Barr Virus (EBV)**

The ISH test for detection of the EBV genome was performed in all tissue specimens using a specific probe for EBV-encoded small RNA-1 (EBER-1, Zhongshan, China) according to the manufacturer’s instructions. EBV-positive NK/T-cell lymphoma and EBV-negative lymphoid tissue were used separately as positive and negative controls.

1. **Bone marrow (BM) examinations by flow cytometry**

Multicolor flow cytometry of BM cells was performed on FACSCalibur flow cytometer (BD Biosciences, US) using fluorochrome-conjugated monoclonal antibodies against HLA-DR, CD117, CD56, BDCA-1, CD9, CD33, CD69, CD11c, partly express CD64dim, CD11b, lake CD123, CD34, CD13, CD15, CD7, CD19, CD14, CD61, CD2, BDCA-2, CD4, CD25, MPO, CD22, Ccd3, bcl-2, CD68, and BDCA-3 (BD Biosciences, US).

1. **Cytogenetic and molecular studies**

Genomic DNA was extracted from formalin-fixed paraffin-embedded tissues by using the QIAamp DNA FFPE Tissue Kit (Qiagen, Germany). Hotspot regions where the point mutations were expected to occur (exon 8, 9, 11, 13 and 17) in the KIT gene were analyzed in the tonsil and BM and amplified by polymerase chain reaction on an ARKTIK thermal cycler (Thermo Scientific, US). The product was sequenced on an ABI 3500 Genetic Analyzer (Applied Biosystems, Waltham Massachusetts, USA). Primers were designed as below:

KIT Exon 8:

F: 5′-GAAGTGAATGTTGCTGAGG-3′

R: 5′-GTGAATTGCAGTCCTTCC-3′

KIT Exon 9:

F: 5′-CTTCCCTTTAGATGCTCTGCTTC-3′

R: 5′-CCTAAACATCCCCTTAAATTGGATT-3′

KIT Exon 11:

F: 5′- GGT GATCTATTTTTCCCTTTCTCCC-3′

R: 5′- TGACATGGAAAGCCCCTGTTTC -3′

KIT Exon 13:

F: 5′- GTATGGTACTGCATGCGCTTG-3′

R: 5′- TAAAAGGCAGCTTGGACACG-3′

KIT Exon 17:

F: 5′- ATGGTTTTCTTTTCTCCTCCAAC-3′

R: 5′- CCTTTGCAGGACTGTCAAGC-3′

Total RNA of BM cells was isolated by using a Qiagen RNeasy mini kit (Qiagen, Germany). Real-time PCR was performed on the ABI PRISM 7500 Sequence Detection System (Applied Biosystems, US) using designed TaqMan probes (Search Biotech Co., Ltd. Beijing). The internal controls, positive controls and negative controls have been set according to the manufacturer. The complete list of 41 leukaemia-related fusion genes tested is follow:

| SIL-TAL1 | E2A-HLF | BCR-ABL | E2A-PBX1 | MLL-AF4 |
| --- | --- | --- | --- | --- |
| TEL-AML1 | MLL-AF9 | AML1-ET0 | TLS-ERG | PML-RARα |
| CBFß-MYH11 | DEK-CAN | NPM-MLF | FIP1L1-PDGFRA | TEL-PDGFRB |
| TEL-ABL | ETV6-PDGFRA | TEL-JAK2 | AML1-MDS1/EVI1 | AML1-MTG16 |
| PLZF-RARα | STAT5b-RARα | NUP98-HoxA13 | NUP98-HoxC11 | NUP98-HoxD13 |
| NUP98-HoxA9 | NUP98-HoxA11 | NUP98-PMX1 | MLL-ENL | MLL-AF10 |
| MLL-ELL | MLL-AF17 | MLL-AF1q | MLL-AF1p | MLL-AF6 |
| MLL-AFX | MLL-SEPT6 | NPM-RARα | FIP1L1-RARα | PRKAR1A-RARα |
| NUMA-RARα |  |  |  |  |
